# Exploring the impact of noise, language familiarity, and experimental settings on emotion recognition

**DOI:** 10.3389/fpsyg.2025.1548975

**Published:** 2025-06-25

**Authors:** Terry Amorese, Marialucia Cuciniello, Anna Alterio, Daniele Pepe, Odette Scharenborg, Gennaro Cordasco, Anna Esposito

**Affiliations:** ^1^Department of Psychology, Università degli Studi della Campania “L. Vanvitelli”, Caserta, Italy; ^2^Multimedia Computing Group, Delft University of Technology, Delft, Netherlands

**Keywords:** speech recognition, vocal emotion recognition, noise, language proficiency, language understanding

## Abstract

**Introduction:**

This work aims to understand the contextual factors affecting speech emotion recognition (SER), more specifically the current research investigates whether the identification of vocal emotional expressions of anger, fear, sadness, joy, and neutrality is affected by three factors: (a) the experimental setting, exploring vocal emotion recognition in both a controlled, soundproof laboratory and a more natural listening environment; (b) the effect of stimuli’s background noise: sentences were presented with three different levels of noise to gradually increase the level of difficulty: one clear (no noise) condition and two noise conditions; (c) language familiarity, since the stimuli comprised Italian sentences, and participants were both native (Italians) and Dutch speakers, who did not know Italian.

**Method:**

Dutch and Italian participants were involved in a vocal emotion recognition task carried out in two different experimental settings (realistic vs. laboratory). The stimuli were vocal utterances from the Italian EMOVO dataset, conveying emotions like anger, fear, sadness, joy, and neutrality, and were presented in three different noise conditions.

**Results:**

Concerning the effect of the experimental setting, even in higher levels of background noise conditions, individuals possess the remarkable ability to discern emotional nuances conveyed through voice. Regarding familiarity with the language, differences in emotion recognition performance between the Italian and Dutch listeners were observed, but the error magnitude was contingent on the emotional categories. Higher noise levels reduced accuracy, but people could still discern emotions, especially prosody.

**Conclusion:**

The study highlighted that emotion recognition is influenced by variables such as listening context, background noise, and language familiarity. These results could be useful for developing robust Speech Emotion Recognition (SER) systems and improving human-computer interaction.

## 1 Introduction

Emotions play a pivotal role in human social functioning (Keltner and Kring, 1998), and the ability to properly perceive and express them is essential for successful human interaction and forms a key aspect of emotional competence (Occhionero and Cicogna, 2008). Deciphering emotions is challenging without context (Kosti et al., 2017; Hess and Hareli, 2016), additionally, the human capacity to interpret and convey emotions is influenced by a multitude of factors, including personal traits, gender, and cultural background (Abbruzzese et al., 2019; Martins et al., 2019; Lorette and Dewaele, 2020). In modern multicultural communities, many individuals frequently communicate using languages different from their mother tongue, often in settings with background noise; the mix of limited language proficiency and a disrupted signal is acknowledged to hinder the processing of sounds and words (Scharenborg et al., 2016, 2018a). However, most psycholinguistic studies investigate emotion perception in a laboratory setting, rather than in a naturalistic setting. Therefore, the aim of this work is to contribute to a deeper understanding of the contextual factors that may impact speech emotion recognition. Understanding how humans interpret emotions from speech is crucial for advancing Speech Emotion Recognition (SER) systems. Combining insights from human emotion perception with advanced machine learning could be helpful to develop SER systems capable of accurately identifying and responding to emotional cues in speech. The presented study represents an extension of a previous one (Scharenborg et al., 2018b) which examined how realistic background noise affects the perception of emotions in a language that listeners do not understand. The researchers aimed to understand this by linking emotion perception to specific acoustic characteristics known to correlate with emotions and by investigating how noise impacts the perception of these characteristics. Dutch students listened to Italian sentences spoken with five different emotions and were asked to identify the emotion conveyed. The sentences were presented in both clean conditions and two levels of babble noise. The authors observed that participants were able to recognize emotions in the unknown language above chance level, even under noisy conditions. Noise had a detrimental effect on emotion perception, but participants still performed better than chance, suggesting that verbal emotion may have universal characteristics. Noise also affected the use of acoustic parameters differently for each emotion category. In the present study, by including Italian participants, the study’s sample was expanded, allowing us to gain a more comprehensive and diverse understanding of the influence of noise on emotion perception. This expansion provided comparative data between native Italian and Dutch listeners, enabling us to analyze whether and how language comprehension affects the ability to recognize emotions under noisy conditions. More specifically, we analyzed the effect of three factors on the recognition of vocal expressions of anger, fear, sadness, joy, and a neutral state, to further understand emotion recognition in multicultural, naturalistic settings:

- The effect of the experimental setting, since vocal emotion recognition was tested in two different listening conditions, a soundproof laboratory context and a realistic listening context.
- The effect of stimuli’s background noise: sentences were presented with three different levels of noise to gradually increase the level of difficulty: one clear (no noise) condition and two noise conditions.
- The effect of the familiarity with a language on vocal emotion recognition, stimuli consisted of Italian sentences, administered to two different groups of participants, native (Italians) and Dutch speakers who do not speak Italian.

## 2 Materials and methods

### 2.1 Participants

Three groups of participants were recruited. The first group (realistic listening context condition) consisted of 37 Dutch participants (mean age = 21.32, SD = ±2.88, 15 females) recruited from the following universities: TU Delft, The Hague University of Applied Sciences, and Leiden University, located in the south-west of the Netherlands. The second group (laboratory listening context condition) was composed of 51 Dutch participants (mean age = 22.15, SD = ±3.14, 25 females), recruited from TU Delft. The third group (realistic listening context condition) consisted of 56 Italian participants (mean age = 22.21, SD = ±2.84, 31 females), recruited from two Universities, Università degli Studi di Napoli Federico II and Università degli Studi Della Campania L. Vanvitelli, both located in the Campania region (south of Italy). None of the participants reported a history of language, speech, or hearing problems. None of the Dutch participants had knowledge of the Italian language. All the participants joined the study on a voluntary basis after signing an informed consent formulated according to the current Italian and European laws about privacy and data protection. The research received the approval of the ethical committee of the Università degli Studi della Campania “Luigi Vanvitelli,” at the Department of Psychology, with the protocol number 25/2017. A detailed overview of participants’ demographic features is provided in Table 1. All participants were university students enrolled in undergraduate or graduate programs in psychology, engineering, or related disciplines. Gender distribution and age statistics (means and standard deviations) are reported below.

**Table 1:** Participants’ demographics by group.

Group	Age range	Mean age	Standard deviation	Females	Males
Dutch (realistic context)	18–31	21.32	±2.88	15	22
Dutch (laboratory context)	18–30	22.15	±3.14	25	26
Italian (realistic context)	18–29	22.21	±2.84	31	25

### 2.2 Stimuli

The stimuli consisted of eight different vocal utterances (4 semantically meaningful and 4 nonsense) conveying anger, fear, sadness, joy, and a neutral state, selected from the Italian EMOVO dataset (Costantini et al., 2014; Giovannella et al., 2009). The Italian EMOVO corpus is a valuable resource for emotion recognition studies. It features 588 emotional utterances in Italian, representing six emotions (anger, fear, sadness, joy, surprise, and disgust) along with a neutral state. These utterances were performed by six professional actors (three men and three women, aged 23–30) using 14 sentences that are emotionally neutral. Nine of these sentences are semantically neutral (e.g., “workers get up early”), while the remaining five are grammatically correct but nonsense. To investigate the effect of background noise on emotion recognition, each sentence was presented in three different noise conditions: a clear condition (no added noise), and two noise conditions with increasing levels of difficulty. The background noise consisted of Italian multi-speaker babble noise, created by overlapping recordings of eight native Italian speakers. This type of noise was selected to simulate realistic background speech environments, such as those encountered in public or social settings. The intensity of the noise was manipulated by adjusting the Signal-to-Noise Ratio (SNR), rather than by altering the semantic content or language of the noise. Specifically, the following SNR levels were used:

+2 dB SNR: a moderate noise condition, in which the speech signal was slightly louder than the background noise.
−5 dB SNR: a high noise condition, in which the background noise was louder than the speech signal.

A third condition with no added noise served as the baseline. All stimuli were normalized for intensity prior to embedding in noise, using a custom-made Praat script (Boersma and Van Heuven, 2001). This procedure ensured consistency across conditions and allowed for a controlled manipulation of auditory difficulty. The stimuli were organized into 12 different experimental lists, each comprising 120 sentences arranged in 3 blocks (i.e., noise condition), the order of which was counterbalanced, as in Scharenborg et al. (2018b). Participants were subsequently randomly assigned to one of the 12 experimental lists. For more details on stimuli selection and noise condition development refer to Scharenborg et al. (2018b).

### 2.3 Procedures

Within the “laboratory listening context condition” participants were tested individually in a sound-treated booth, while for the “realistic listening context condition” participants were tested in a quiet cafeteria within a library or in a university study room. These settings, although generally quiet, were natural environments that could still experience occasional disturbances, such as the noise from a cafeteria’s coffee machine or the movement of nearby individuals, potentially affecting participants’ attention. Before starting the experiment, participants completed a consent form and questionnaire to assess exclusion criteria (above described in Section 2.1). Instructions emphasized the importance of focusing solely on the emotional content of the presented sentences, disregarding textual context and background noise. The stimuli were presented through headphones at a comfortable sound level. Participants then identified the conveyed emotion by selecting from options including joy, anger, fear, sadness, neutral, and an ‘I do not know’ option using a labeled keyboard. To prevent fatigue and attentional decline, designated breaks were included, allowing participants to resume at their discretion.

## 3 Data analysis and results

The data collected were analyzed through three separate repeated measures ANOVAs conducted using SPSS 21 IBM software. The first analysis was carried out with the aim of comparing emotion recognition performances of Dutch participants in two different listening conditions: laboratory vs. realistic contexts. For this analysis participants’ gender (only binary labels are available: F and M) and the context (laboratory and realistic) were considered as between subject variables, while the proposed emotion (joy, neutral, fear, anger, and sadness) and the noise condition (clear, +2 dB and −5 dB) were considered as within subject variables. The significance level was set at *α* < 0.05 and differences among means were assessed through Bonferroni’s *post-hoc* tests. The second analysis was carried out with the aim of comparing emotion recognition performances of native Italians and Dutch speakers, in realistic listening contexts. For this analysis participants’ gender (F and M) and the country of origin (Italians and Dutch) were considered as between subject variables, while the proposed emotion (joy, neutral, fear, anger, and sadness) and the noise condition (clear, +2 dB and −5 dB) were considered as within subject variables. The significance level was set at *α* < 0.05 and differences among means were assessed through Bonferroni’s *post-hoc* tests. The third analysis was carried out with the aim of testing the effect of language understanding on emotion recognition and to deeply understand some results obtained from the second analysis (which compared Italian and Dutch speakers), regardless of the noise condition. For this analysis participants’ gender (F and M) and the country of origin (Italians and Dutch) were considered as between subject variables, while the proposed emotion (joy, neutral, fear, anger, and sadness) and the meaning condition (meaningful vs. meaningless sentences) were considered as within subject variables. The significance level was set at *α* < 0.05 and differences among means were assessed through Bonferroni’s *post-hoc* tests. In each conducted analysis the dependent variable was represented by emotions’ mean recognition scores.

### 3.1 Laboratory vs. realistic context

**Figure 1:**
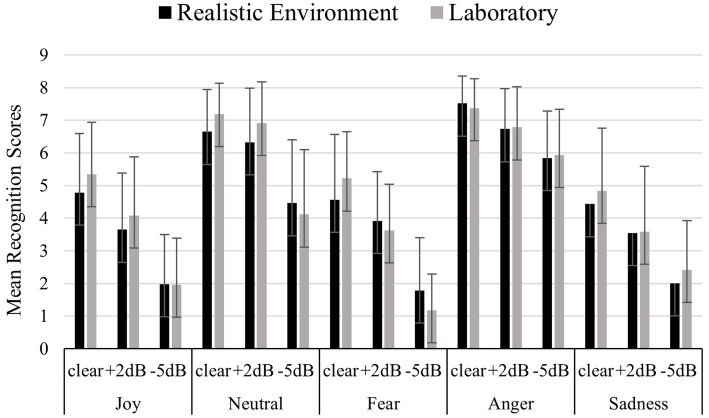
Mean recognition scores for each emotional category and noise condition (clear, +2 dB, −5 dB), comparing Dutch participants tested in realistic versus laboratory environments. Each emotion was presented through 8 stimuli per noise condition, with total recognition scores ranging from 0 to 8. The y-axis displays the mean recognition scores, which reflect the average number of correctly recognized stimuli per condition.

Figure 1 shows mean recognition scores (for each emotional category and for each noise condition) comparing each group, namely Dutch participants who were tested in realistic vs. laboratory environment. Mean recognition scores were calculated as follows: each emotion was presented through 8 stimuli for each noise condition (clear, +2 dB and −5 dB). For each condition, the recognition scores were added, obtaining a total recognition score ranging from 0 to 8. On this total recognition scores were calculated means presented on the y-axis of the figure, and these means can therefore vary between 0 and 8 for each condition. No significant effects of participants’ gender [F (1,84) = 1.170, *p* = 0.283] and listening conditions (laboratory vs. realistic context) [F (1,84) = 0.604, *p* = 0.439] were found. A significant difference in the recognition of emotional categories was found [F (4,336) = 206.251, *p* <<0.01]. Bonferroni’s *post-hoc* tests revealed that Anger (mean = 6.705) and Neutral (mean = 5.972) were the emotional categories better recognized (*p* <<0.01) compared to Joy (mean = 3.625), Fear (mean = 3.383) and Sadness (mean = 3.503). A significant effect of noise condition was found [F (2,168) = 468.655, *p* <<0.01], with lower accuracies for harder noise conditions according to the Bonferroni’s *post-hoc* tests (clear mean = 5.817; +2 dB mean = 4.917; −5 dB mean = 3.179, *p* <<0.01). A significant interaction [F (2,168) = 3.712, *p* = 0.026] emerged between noise condition and listening conditions (laboratory vs. realistic context). Bonferroni’s *post-hoc* tests were carried out for each single factor (noise condition and listening conditions). These tests revealed the following:

a) Concerning the listening conditions (laboratory vs. realistic context): no significant differences emerged according to stimuli’s noise condition.
b) Concerning noise condition: it was observed that in both laboratory and realistic conditions the more noise, the less accurately the stimuli were recognized (clear laboratory mean = 5.997; +2 dB laboratory mean = 5.002; −5 dB laboratory mean = 3.122, *p* <<0.01) (clear realistic mean = 5.638; +2 dB realistic mean = 4.832; −5 dB realistic mean = 3.237, *p* <<0.01).

A significant interaction [F (8,672) = 9.682, *p* <<0.01] emerged between noise condition (clear, +2 dB, and −5 dB) and the emotional category (joy, neutral, fear, anger, and sadness). Bonferroni’s *post-hoc* tests carried out for each single factor (noise condition and emotional category) revealed:

a) Concerning noise condition: for each emotional category the more noise, the less accurately the stimuli were recognized [(Joy clear = 5.084, Joy +2 dB = 3.842, Joy −5 dB = 1.951, *p* <<0.01) (Fear clear = 4.867, Fear +2 dB = 3.738, Fear −5 dB = 1.545, *p* <<0.01) (Anger clear = 7.455, Anger +2 dB = 6.785, Anger −5 dB = 5.902, *p* <<0.01) (Sadness clear = 4.691, Sadness +2 dB = 3.605, Sadness −5 dB = 2.213, *p* <<0.01). The only exception was represented by Neutral, in which no differences between the no noise condition were observed (mean = 6.989) and +2 dB (mean = 6.642), while worse performances for the −5 dB noise condition were observed (mean = 4.286, *p* <<0.01).
b) Concerning the emotional categories: when no noise was present, Anger (mean = 6.989) and Neutral (mean = 7.455) were better recognized (*p* < 0.05) followed by Joy (mean = 5.084), Fear (mean = 4.867) and Sadness (mean = 4.691). The same occurred when noise condition corresponded to +2 dB, with Anger (mean = 6.759) and Neutral (mean = 6.642) better recognized (*p* <<0.01) compared to Joy (mean = 3.842), Fear (mean = 3.738) and Sadness (mean = 3.605) and when noise condition corresponded to −5 dB with Anger (mean = 5.902) and Neutral (mean = 4.286) better recognized (*p* <<0.01) compared to Joy (mean = 1.951), Fear (mean = 1.545) and Sadness (mean = 2.213).

A difference between listening conditions (laboratory vs. realistic context) emerged thanks to a significant triple interaction among noise condition, emotion categories and listening conditions [F (8,672) = 2.280, *p* = 0.021]. Bonferroni *post-hoc* test revealed that this is due to the emotional category of Fear, that when presented with −5 dB noise was more accurately decoded in the realistic context (mean = 1.914) compared to the laboratory context (mean = 1.176, *p* = 0.010).

### 3.2 Italian vs. Dutch listeners

**Figure 2:**
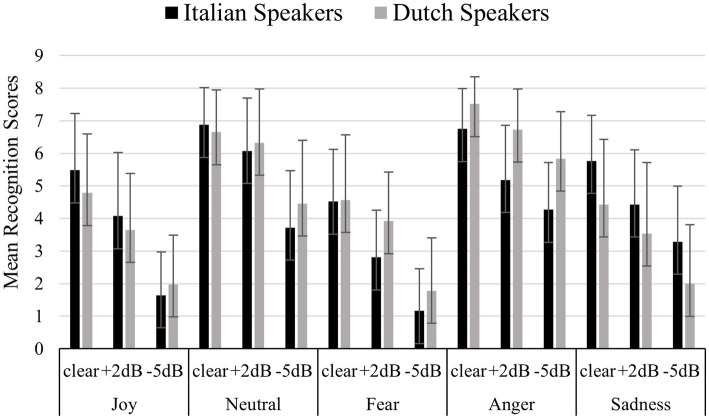
Mean recognition scores for each emotional category and noise condition (clear, +2 dB, −5 dB), comparing Italian native speakers and Dutch speakers in realistic listening contexts. Each emotion was presented through 8 stimuli per noise condition, yielding total recognition scores ranging from 0 to 8. The y-axis displays the mean recognition scores, representing the average number of correctly recognized stimuli per condition.

Figure 2 shows mean recognition scores (for each emotional category and for each noise condition) comparing Italian native speakers and Dutch speakers in realistic listening contexts. Mean recognition scores were calculated as following: each emotion was presented through 8 stimuli for each noise condition (clear, +2 dB and −5 dB). For each condition, the recognition scores were added, obtaining a total recognition score ranging from 0 to 8. On this total recognition scores were calculated means presented on the y-axis of the figure, and these means can therefore vary between 0 and 8 for each condition. A significant effect of participants’ gender emerged [F (1,89) = 4.416, *p* = 0.038]. Bonferroni *post-hoc* tests revealed female participants more accurately recognize the emotions (mean = 4.652) than the male participants (mean = 4.293, *p* = 0.038). No significant effects of the country of origin (Italians and Dutch speakers) were observed [F (1,89) = 1.263, *p* = 0.264]. A significant difference in the recognition of emotional categories emerged [F (4,356) = 171.203, *p* <<0.01]. Bonferroni’s *post-hoc* tests revealed that Anger (mean = 6.037) and Neutral (mean = 5.709) were better recognized (*p* <<0.01) than Joy (mean = 3.581), Fear (mean = 3.117) and Sadness (mean = 3.919). A significant effect of noise condition was found [F (2,178) = 343,934, *p* <<0.01], with lower accuracies for more noise according to the Bonferroni’s *post-hoc* tests (clear mean = 5.750; +2 dB mean = 4.655; −5 dB mean = 3.012, *p* <<0.01). A significant interaction [F (2,178) = 6.008, *p* = 0.003] was found between noise condition (clear, +2 dB, and −5 dB) and participants’ country of origin (Italians and Dutch speakers). Bonferroni’s *post-hoc* tests were carried out for each single factor (noise condition and country of origin), which showed:

a) Concerning participants’ country of origin: native Italians showed worse recognition performances (mean = 2.788) compared to the Dutch speakers (mean = 3.237, *p* = 0.037) for the −5 dB condition.
b) Concerning the noise condition: for both Italians native speakers and Dutch speakers the more noise, the less accurately the stimuli were recognized (clear Dutch mean = 5.638; +2 dB Dutch mean = 4.832; −5 dB Dutch mean = 3.237, *p* <<0.01) (clear Italians mean = 5.862; +2 dB Italians mean = 4.479; −5 dB Italians mean = 2.788, *p* <<0.01).

A significant interaction [F (4,356) = 20.483, *p* <<0.01] emerged between participants’ country of origin (Italians native speakers and Dutch speakers) and the emotional category (joy, neutral, fear, anger, and sadness). Bonferroni’s *post-hoc* tests were carried out for each single factor (country of origin and emotional category), which revealed:

a) Concerning participants’ country of origin: for Fear and Anger, Dutch speakers showed better performances (Fear mean = 3.427; Anger mean = 6.711) than the Italian native speakers (Fear mean = 2.807, *p* = 0.011; Anger mean = 5.364, *p* <<0.01), while for Sadness, Italian native speakers showed better performance (mean = 4.451) than Dutch speakers (mean = 3.386, *p* <<0.01).
b) Concerning the emotional category: Dutch speakers better recognized Anger (mean = 6.711) and Neutral (mean = 5.866) (*p* <<0.01) compared to Joy (mean = 3.454), Fear (mean = 3.427) and Sadness (mean = 3.386). Italian native speakers better recognized Anger (mean = 5.364) and Neutral (mean = 5.553) and Sadness (mean = 4.451) compared to Joy (mean = 3.708) and Fear (mean = 2.807).

A significant interaction [F (8,712) = 5.109, *p* <<0.01] emerged between noise condition (clear, +2 dB, and −5 dB) and the emotional category. Bonferroni’s *post-hoc* tests were carried out for each single factor (noise condition and emotional category), which showed:

a) Concerning noise condition: for each emotional category the more noise, the less accurately the stimuli were recognized [(Joy clear = 5.154, Joy +2 dB = 3.810, Joy −5 dB = 1.779, *p* <<0.01) (Neutral clear = 6.837, Neutral +2 dB = 6.199, Neutral −5 dB = 4.092) (Fear clear = 4.500, Fear +2 dB = 3.323, Fear −5 dB = 1.527, *p* <<0.01) (Anger clear = 7.127, Anger +2 dB = 5.936, Anger −5 dB = 5.049, *p* <<0.01) (Sadness clear = 5.133, Sadness +2 dB = 4.008, Sadness −5 dB = 2.615, *p* <<0.01).
b) Concerning the emotional categories: in the no noise condition, Anger (mean = 7.127) and Neutral (mean = 6.837) were recognized best (*p* <<0.01) followed by Joy (mean = 5.154), Sadness (mean = 5.133) and Fear (mean = 4.500). The same trend was observed for the +2 dB condition, with Anger (mean = 5.936) and Neutral (mean = 6.199) better recognized (*p* <<0.01) than Joy (mean = 5.154), Fear (mean = 3.323) and Sadness (mean = 4.008), and the −5 dB condition with Anger (mean = 5.049) and Neutral (mean = 4.092) better recognized (*p* <<0.01) compared to Joy (mean = 1.779), Fear (mean = 1.527) and Sadness (mean = 2.615).

### 3.3 Meaningful vs. nonsense sentences

**Figure 3:**
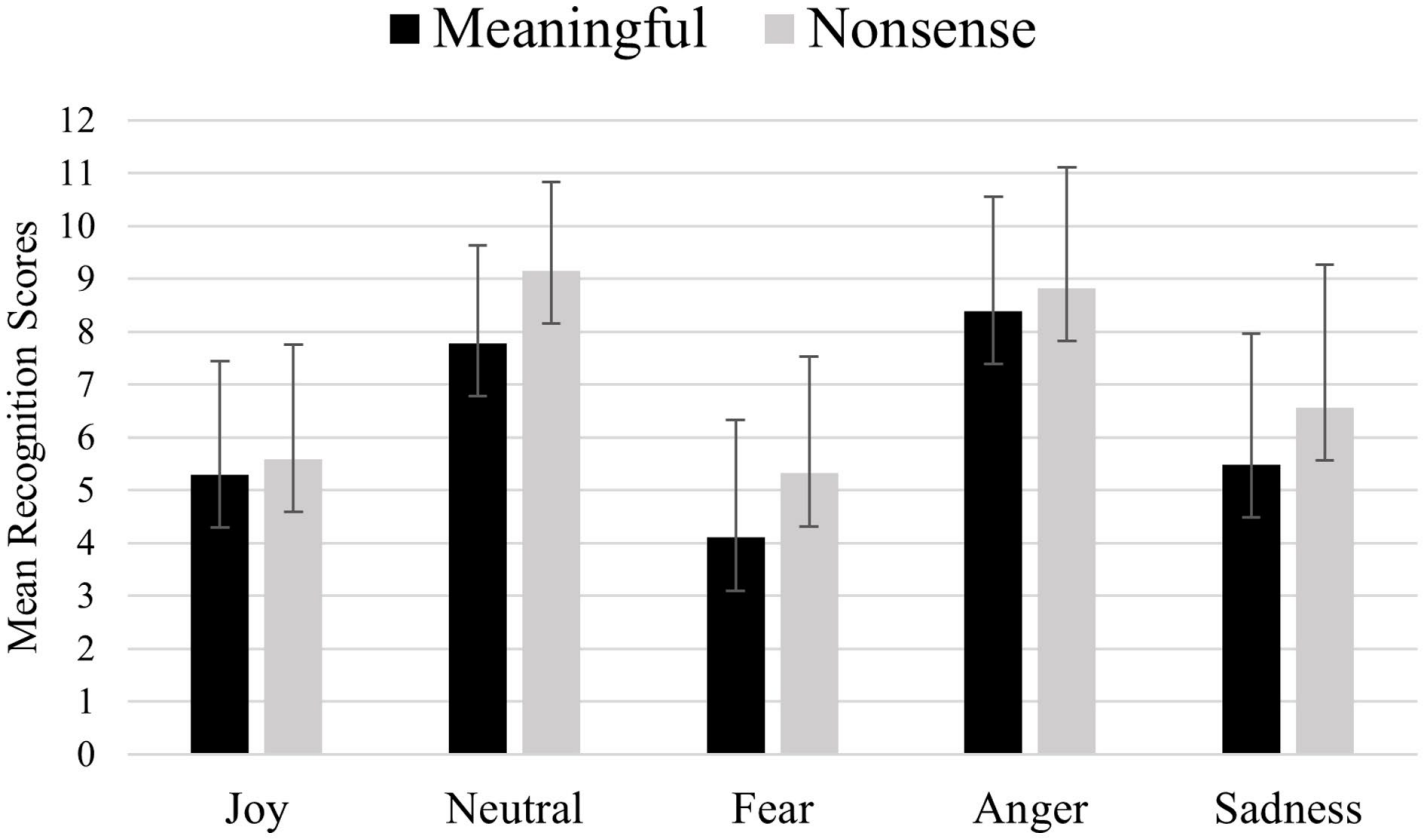
Mean recognition scores for each emotional category, comparing semantically meaningful and nonsense sentences. Each emotion was presented through 8 stimuli per noise condition (clear, +2 dB, −5 dB), comprising 4 meaningful and 4 nonsense sentences, resulting in 12 meaningful and 12 nonsense stimuli per emotion. Total recognition scores ranged from 0 to 12. The y-axis displays the mean recognition scores, representing the average number of correctly recognized stimuli for each emotion.

Figure 3 shows (for each emotional category) mean recognition scores of meaningful and nonsense sentences. Mean recognition scores were calculated as following: each emotion was presented through 8 stimuli (4 semantically meaningful and 4 nonsense) for each noise condition (clear, +2 dB and −5 dB), for a total of 12 semantically meaningful and 12 nonsense sentences. For each emotion, the recognition scores were added, obtaining a total recognition score ranging from 0 to 12. On this total recognition scores were calculated means presented on the y-axis of the figure, and these means can therefore vary between 0 and 12 for each emotion. No significant effects of participants’ gender [F (1,89) = 2.579, *p* = 0.112] and country [F (1,89) = 0.414, *p* = 0.522] were found. A significant difference was observed [F (1,89) = 38.780, *p* <<0.01] in the recognition of meaningful and nonsense sentences. Bonferroni’s *post-hoc* tests revealed that this was due to nonsense sentences (mean = 7.030) which were better decoded compared to meaningful sentences (mean = 6.273). A significant interaction [F (4,356) = 4.033, *p* = 0.003] emerged between stimuli’s meaning condition (meaningful vs. nonsense) and the emotional categories. Bonferroni’s *post-hoc* tests showed that Neutrality, Fear and Sadness were better decoded when expressed through nonsense sentences (nonsense neutrality mean = 9.123; nonsense fear mean = 5.387; nonsense sadness mean = 6.369) than when expressed through meaningful sentences (meaningful neutrality mean = 7.921; meaningful fear mean = 4.258; meaningful sadness mean = 5.404).

## 4 Discussion and conclusion

The present study investigated how recognition of emotional expressions conveyed through vocal stimuli, can be affected by important variables, specifically the Experimental setting, Background Noise, and Language Familiarity.

The main result of our first analysis is that the influence of the listening context (laboratory vs. naturalistic environment) was only found for the recognition of Fear in the SNR −5 dB condition. For the other emotions and noise conditions, no significant differences were observed. Although laboratory findings do not always generalize to naturalistic settings, our results suggest that laboratory findings on emotion perception generalize at least to relatively quiet, naturalistic settings. Our results suggest that even in higher levels of background noise, people can still discern the emotional state from the voice. A possible explanation of this is that the recognition of prosody, the intonation and rhythm of speech (fundamental for vocal emotion recognition), tends to remain significantly better than chance even in highly noisy conditions (Van Zyl and Hanekom, 2011; Morgan, 2021). According to Van Zyl and Hanekom (2011) the ability to comprehend speech in challenging listening environments is greatly aided by the redundancy inherent in the speech signal, and prosody, significantly contributes to this redundancy. It was also observed that two emotional categories were better recognized than the others, namely anger and neutral. This result is also confirmed by other studies (Scharenborg et al., 2018b; Pell et al., 2009). Moreover, the background noise condition was found to influence emotion processing: higher noise conditions led to less accurate emotion recognition (in line with Scharenborg et al., 2016, 2018b). Recognizing emotions in noisy environments is essential for several reasons, especially in fields like psychology, communication, and human-computer interaction. Firstly, effective communication often depends on understanding emotional cues, which can be difficult in noisy settings. Accurately recognizing emotions helps maintain clear and effective interactions, reducing misunderstandings. Additionally, in noisy environments such as workplaces or public spaces, detecting emotional distress or urgency in someone’s voice is crucial for safety and prompt response. Improving emotion recognition in noise is vital for developing robust speech recognition systems and virtual assistants that can operate effectively in real-world conditions. Social interactions frequently occur in noisy environments (e.g., parties, restaurants), and the ability to accurately perceive emotions in these settings is important for maintaining social bonds and empathy. Lastly, noise can increase cognitive load and stress, making it harder to process emotional information. Understanding how to mitigate these effects can enhance overall cognitive and emotional functioning (Weninger et al., 2013; Zhang et al., 2023).

The main result of our second analysis is that there are significant differences in emotion recognition performance between the native Italian listeners and the Dutch listeners without any knowledge of Italian. This difference particularly emerged for the worst listening conditions, i.e., when stimuli were presented with an SNR of −5 dB. Surprisingly, the Dutch listeners outperformed the Italian listeners. To investigate whether this might be attributed to an effect of understanding the language, an additional analysis was carried out comparing the effect of meaningful and nonsense sentences. This analysis showed a significant effect of the type of sentence on emotional recognition, i.e., meaningless sentences were recognized more accurately than meaningful ones (*p* <<0.01). However, no significant difference was observed between the Italian and the Dutch listeners. The effect of understanding the language thus does not explain the observed difference between the Italians and Dutch for the worst listening conditions and needs further research. The observed difference between meaningful and nonsense sentences should likely be attributed to the speakers rather than the listeners. The speakers might have conveyed the emotions more clearly in the nonsense sentences than the semantically meaningful sentences as the former is cognitively free of meaning. There thus would not have been any potential incongruence between the meaning of the sentence and the emotion, which might have been the case for the meaningful sentences. However, these results contribute to the existing body of evidence, showing consistent high levels of accuracy in tasks involving the recognition of emotional expressions in languages other than an individual’s native language (Scherer et al., 2001; Thompson and Balkwill, 2006). Furthermore, there were differences found in the recognition of the different emotion categories. For Fear and Anger, Dutch speakers showed better performances than Italians (native) speakers, while Italians better recognized Sadness compared to the Dutch participants. These results are in contrast with the “language expertise hypothesis” (Esposito, 2009) which proposes that people are better at recognizing emotional expressions in audio stimuli when they are asked to label them using their native language since they have a deep understanding of the specific patterns unique to their language; this familiarity should enable them to interpret emotional information more accurately compared to when they are using a non-native language. However, the results are in line with other studies (Parada-Cabaleiro et al., 2017; Silva et al., 2016) suggesting that while there are universally recognizable vocal cues for conveying basic emotions, not all affective states, follow this universal recognition, and that deciphering emotional involves a blend of cues that are universally understood as well as those specific to particular culture. Participants in psycholinguistic studies are often women, and gender is typically not balanced among the participant groups, the underlying assumption (often) being that speech perception is similar for female and male listeners (Baxter et al., 2003; Clements et al., 2006). However, some studies (Walla et al., 2001; Strelnikov et al., 2009) in contrast, highlighted clear gender differences in speech perception. Although our participant group was also not perfectly balanced for gender, our second analysis showed an effect of the gender of the listener: overall female participants recognized the emotional categories more accurately than males, which is in line with other studies that investigated the role of gender in emotion recognition (Demenescu et al., 2014; Lausen and Schacht, 2018). However, results from research examining gender disparities in the recognition of emotions fluctuate, contingent upon the specific types of emotional category considered and the sensory channels exploited to present stimuli (Lambrecht et al., 2014).

Even if it is well known that vocal expression of emotions, or in other words emotional states conveyed through a single channel, convey the same amount of information as the combined channels (as for instance visual and auditory) (Esposito, 2007), future studies could replicate the experiment by adding a condition in which emotional expressions are also conveyed through facial expressions and/or gestures, so as to test what happens for listeners unfamiliar with a language when the visual channel is also involved in the emotional recognition process. Future research may also focus on testing participants from different age groups (for instance, children and seniors) to evaluate age-related differences in vocal emotion recognition.

One limitation of the present study is the homogeneity of participant samples in terms of both age and educational background. All participants were young adults and university students. This may introduce age- and education-related biases, potentially limiting the generalizability of the findings to older populations or individuals with different educational experiences. Future research should include participants from a broader age range and more diverse educational backgrounds to explore how these factors may influence vocal emotion recognition.

In conclusion, laboratory results on emotion recognition in background noise for native speakers of the language and those that do not know the language generalize well to naturalistic settings. Moreover, an effect of familiarity of the language was found, native speakers and those that do not speak the language showed differential effects for background noise, with lower accuracies for the native speakers for the hardest noise condition. Hopefully these findings will be useful in the field of Human-Computer Interaction, and in particular in the implementation of Speech Emotion Recognition (SER) systems, which are thought to identify different emotions expressed by speakers through the analysis and classification of key features extracted from speech signals. Considering that, understanding how humans process emotions from speech is essential to analyze how machines recognize and correlate emotional aspects of speech signals, since the aim of speech processing research is to develop machines capable of executing tasks such as automatic speech recognition, speech synthesis, and speaker recognition, among others, with proficiency comparable to humans.

## Data Availability

The raw data supporting the conclusions of this article will be made available by the authors, without undue reservation.

## References

[ref1] Abbruzzese L. Magnani N. Robertson I. H. Mancuso M. (2019). Age and gender differences in emotion recognition. Front. Psychol. 10:2371. doi: 10.3389/fpsyg.2019.02371, PMID: 31708832 PMC6819430

[ref2] Baxter L. C. Saykin A. J. Flashman L. A. Johnson S. C. Guerin S. J. Babcock D. R.. (2003). Sex differences in semantic language processing: a functional MRI study. Brain Lang. 84, 264–272. doi: 10.1016/S0093-934X(02)00549-7, PMID: 12590915

[ref3] Boersma P. Van Heuven V. (2001). Speak and unSpeak with PRAAT. Glot Int. 5, 341–347.

[ref4] Clements A. M. Rimrodt S. L. Abel J. R. Blankner J. G. Mostofsky S. H. Pekar J. J.. (2006). Sex differences in cerebral laterality of language and visuospatial processing. Brain Lang. 98, 150–158. doi: 10.1016/j.bandl.2006.04.007, PMID: 16716389

[ref5] Costantini G. Iaderola I. Paoloni A. Todisco M.. (2014). EMOVO corpus: an Italian emotional speech database. In Proceedings of the ninth international conference on language resources and evaluation (LREC’14) (3501–3504). European Language Resources Association (ELRA).

[ref6] Demenescu L. R. Mathiak K. A. Mathiak K. (2014). Age- and gender-related variations of emotion recognition in pseudowords and faces. Exp. Aging Res. 40, 187–207. doi: 10.1080/0361073X.2014.882210, PMID: 24625046

[ref7] Esposito A. (2007). “The amount of information on emotional states conveyed by the verbal and nonverbal channels: some perceptual data” in Progress in nonlinear speech processing. Eds. Stylianou Y. Faundez-Zanuy M. Esposito A. (Berlin, Heidelberg: Springer), 249–268.

[ref8] Esposito A. (2009). The perceptual and cognitive role of visual and auditory channels in conveying emotional information. Cogn. Comput. 1, 268–278. doi: 10.1007/s12559-009-9017-8

[ref9] Giovannella C. Conflitti D. Santoboni R. Paoloni A.. (2009). Transmission of vocal emotion: do we have to care about the listener? The case of the Italian speech corpus EMOVO. In 2009 3rd international conference on affective computing and intelligent interaction and workshops (1–6). IEEE.

[ref10] Hess U. Hareli S. (2016). “The impact of context on the perception of emotions” in The expression of emotion: philosophical, psychological, and legal perspectives. Eds. Abell C. Smith J. (Cambridge University Press), 199–218.

[ref11] Keltner D. Kring A. M. (1998). Emotion, social function, and psychopathology. Rev. Gen. Psychol. 2, 320–342. doi: 10.1037/1089-2680.2.3.320

[ref12] Kosti R. Alvarez J. M. Recasens A. Lapedriza A. (2017) Emotion recognition in context. In Proceedings of the IEEE Conference on Computer Vision and Pattern Recognition (pp. 1667–1675)

[ref13] Lambrecht L. Kreifelts B. Wildgruber D. (2014). Gender differences in emotion recognition: impact of sensory modality and emotional category. Cognit. Emot. 28, 452–469. doi: 10.1080/02699931.2013.837378, PMID: 24151963

[ref14] Lausen A. Schacht A. (2018). Gender differences in the recognition of vocal emotions. Front. Psychol. 9:882. doi: 10.3389/fpsyg.2018.00882, PMID: 29922202 PMC5996252

[ref15] Lorette P. Dewaele J. M. (2020). Emotion recognition ability across different modalities: the role of language status (L1/LX), proficiency and cultural background. Appl. Linguist. Rev. 11, 1–26. doi: 10.1515/applirev-2017-0015

[ref16] Martins A. T. Ros A. Valério L. Faísca L. (2019). Basic emotion recognition according to clinical personality traits. Curr. Psychol. 38, 879–889. doi: 10.1007/s12144-017-9661-1

[ref17] Morgan S. D. (2021). Comparing emotion recognition and word recognition in background noise. J. Speech Lang. Hear. Res. 64, 1758–1772. doi: 10.1044/2021_JSLHR-20-00153, PMID: 33830784

[ref18] Occhionero M. Cicogna P. C. (2008). Psicologia Generale. Storia della psicologia scientifica. Bologna: Il Mulino.

[ref19] Parada-Cabaleiro E. Baird A. Batliner A. Cummins N. Hantke S. Schuller B. (2017). “The perception of emotions in noisified nonsense speech.” In Proceedings of Interspeech 2017. (Stockholm, Sweden: International Speech Communication Association (ISCA)).

[ref20] Pell M. D. Monetta L. Paulmann S. Kotz S. A. (2009). Recognizing emotions in a foreign language. J. Nonverbal Behav. 33, 107–120. doi: 10.1007/s10919-008-0065-7

[ref21] Scharenborg O. Coumans J. M. van Hout R. (2018a). The effect of background noise on the word activation process in nonnative spoken-word recognition. J. Exp. Psychol. Learn. Mem. Cogn. 44, 233–249. doi: 10.1037/xlm0000441 28782967

[ref22] Scharenborg O. Kakouros S. Koemans J.. (2018b). The effect of noise on emotion perception in an unknown language. In International conference on speech prosody (364–368). International Speech Communication Association (ISCA).

[ref23] Scharenborg O. E. Kolkman E. Kakouros S. Post B. M. B. (2016). The effect of sentence accent on non-native speech perception in noise. In Proceedings of Interspeech 2016. (San Francisco, USA: International Speech Communication Association (ISCA)).

[ref24] Scherer K. R. Banse R. Wallbott H. G. (2001). Emotion inferences from vocal expression correlate across languages and cultures. J. Cross-Cult. Psychol. 32, 76–92. doi: 10.1177/0022022101032001009

[ref25] Silva W. D. Barbosa P. A. Abelin Å. (2016). Cross-cultural and cross-linguistic perception of authentic emotions through speech: an acoustic-phonetic study with Brazilian and Swedish listeners. DELTA 32, 449–480. doi: 10.1590/0102-445003263701432483

[ref26] Strelnikov K. Rouger J. Lagleyre S. Fraysse B. Deguine O. Barone P. (2009). Improvement in speech-reading ability by auditory training: evidence from gender differences in normally hearing, deaf and cochlear implanted subjects. Neuropsychologia 47, 972–979. doi: 10.1016/j.neuropsychologia.2008.10.017, PMID: 19022268

[ref27] Thompson W. F. Balkwill L. L. (2006). Decoding speech prosody in five languages. (Semiotica, De Gruyter), 407–424.

[ref28] Van Zyl M. Hanekom J. J. (2011). Speech perception in noise: a comparison between sentence and prosody recognition. J. Hear. Sci. 1, 54–56.

[ref29] Walla P. Hufnagl B. Lindinger G. Deecke L. Lang W. (2001). Physiological evidence of gender differences in word recognition: a magnetoencephalographic (MEG) study. Cogn. Brain Res. 12, 49–54. doi: 10.1016/S0926-6410(01)00028-3, PMID: 11489608

[ref30] Weninger F. Eyben F. Schuller B. W. Mortillaro M. Scherer K. R. (2013). On the acoustics of emotion in audio: what speech, music, and sound have in common. Front. Psychol. 4:292. doi: 10.3389/fpsyg.2013.00292, PMID: 23750144 PMC3664314

[ref31] Zhang M. Zhang H. Tang E. Ding H. Zhang Y. (2023). Evaluating the relative perceptual salience of linguistic and emotional prosody in quiet and noisy contexts. Behav. Sci. 13:800. doi: 10.3390/bs13100800, PMID: 37887450 PMC10603920

